# Barriers to the delivery of contextualised care in UK small animal veterinary practice

**DOI:** 10.18849/ve.v11i2.752

**Published:** 2026-06-03

**Authors:** Sally Everitt, Andréa Taylor, Ashley Doorly, Katherine Cropley, Allison Osterberg, Katie Mantell

**Affiliations:** RCVS Knowledge United Kingdom; Kaleidoscope Health and Care, United Kingdom

**Keywords:** BARRIERS, CONTEXTUALISED CARE, PET OWNERSHIP, SMALL ANIMAL, VETERINARY PRACTICE

## Abstract

**Objective:**

To identify the main barriers to the delivery of contextualised care in UK small animal veterinary practice, and to understand how these are experienced in practice.

**Background:**

The term contextualised care was introduced in the veterinary context in a published article by members of Veterinary Humanities UK. Although the term has been widely adopted there has been no explicit research into contextualised care in practice.

**Evidentiary value:**

This paper presents the first empirical evidence using quantitative and qualitative data on the barriers to contextualised care as perceived by over 400 veterinary professionals and over 700 dog and cat owners. This evidence should help veterinary teams to identify potential barriers to the delivery of contextualised care.

**Methods:**

The study used a mixed methods approach analysing data from questionnaires, interviews, and focus groups.

**Results:**

The most frequently reported barriers related to lack of continuity of care and financial constraints, but the research also found that fear of regulatory scrutiny, missed or incorrect diagnoses, and client complaints could act as a barrier to adapting care to the individual animal, owner, and context. The study found some variation in the barriers encountered based on experience, role, and practice type.

**Conclusion:**

Overcoming the barriers to contextualised care will require support at all levels, including from professional bodies, educators, and those with leadership positions in veterinary practice.

**Application:**

This research should help veterinary teams to overcome potential barriers to the delivery of contextualised care.

## Introduction

The term ‘contextualised care’ was introduced in the veterinary context to describe an approach in which ‘different treatment pathways are able to offer equally acceptable patient journeys in different contexts’ and ‘that is intentionally shaped by the aims, knowledge, experiences and circumstances of individual animal caregivers and veterinary professionals, acknowledging the wider contexts of each clinical encounter, to deliver the most appropriate welfare-focused care for every animal’ (Skipper et al., 2021, 2024).

The idea of adapting care to the individual circumstances of patients and their owners, as well as the context in which the care is provided, may previously have been described as the ‘art of veterinary practice’ (May, 2015; Rollin, 2020). However, there are a number of reasons why it may now be more difficult to adopt this approach, including: advances in the range of diagnostic tests and treatments available, with associated higher costs leading to concerns about affordability and accessibility; changes in the way that veterinary services are provided; and the fact that pets are being increasingly seen as family members, leading to altered expectations for veterinary care (Corr et al*.*, 2024).

The discussion in the UK has contrasted contextualised care with gold standard care, that is care associated with intensive and technologically advanced approaches, which is assumed to be the preferred option*, *in the belief that it is most likely to result in the best outcomes (Skipper et al., 2021; Englar, 2023). Concerns have also been raised that advances in veterinary medicine may have led to a situation where some available diagnostic investigations and treatments are unaffordable to owners and not in the best interests of the animal (Grimm et al*.*, 2018; Quain et al., 2021).

Discussions regarding contextualised care have also been brought into sharper focus because of the Competition and Markets Authority (CMA) market investigation into veterinary services for household pets, with a number of organisations referencing contextualised care in their responses (CMA, 2024). Despite the widespread use of the term, there is currently little evidence into how contextualised care is understood and delivered in practice. To answer these questions, RCVS Knowledge partnered with Kaleidoscope Health and Care to conduct a research project to explore how contextualised care is understood, experienced, and applied within UK small animal veterinary practice, what barriers prevent its delivery, what it looks like when done well, and what changes might be needed to support this approach. The research focused on veterinary care for dogs and cats as these make up the majority of small animal consultations and have been most impacted by the changes in the delivery of veterinary services (Sánchez-Vizcaíno et al*.*, 2017).

This paper reports specifically on the main barriers to the delivery of contextualised care identified by veterinary professionals working in small animal practice and dog and cat owners.

## Methods

### Study design and data collection tools

This research employed a mixed-methods approach to capture both the breadth and depth of stakeholder experiences including both quantitative data (questionnaires) and qualitative data (focus groups, interviews and free-text questionnaire responses) from those working in veterinary practice and from dog and cat owners.

Development of the research framework and data collection instruments was informed by findings from a review of relevant literature as well as discussions at the ‘National Forum on Contextualised Care: Shaping the Strategy’, held in London in February 2025. At this event more than 70 participants, including veterinary surgeons, Registered Veterinary Nurses (RVNs), receptionists, educators, regulators, leaders of veterinary associations, leaders from charity and rescue settings, and pet owners participated in discussions focused on the relevance of contextualised care, and the barriers and enablers to its delivery.

The data collection tools included two surveys, one for veterinary professionals (veterinary surgeons and RVNs in clinical practice) and one for owners of dogs and cats. These composed multi-select, multiple-choice, and Likert-type questions which collected quantitative data, and open-text fields which captured qualitative data. The draft surveys were piloted with a subset of the target populations, which informed refinements to question phrasing, response options, and survey logic prior to full launch.

Interview guides were tailored to specific roles (e.g. veterinary surgeon, RVN, practice administrator, veterinary educator) but all followed a similar structure based on the research questions, with flexibility for probing and exploration of emergent themes. The focus group guide was also framed by the research questions and aimed to explore pet owners’ experiences and perspectives of obtaining appropriate veterinary care.

#### Participants and recruitment

Both surveys were hosted on the Alchemer online survey platform and were active for four weeks in April 2025. Response rates were monitored throughout the data collection period.

The inclusion criteria for the veterinary professional survey were qualified veterinary surgeons and RVNs, over 18 years of age, currently practising in the UK who provide clinical care to dogs and/or cats. The survey was promoted through multiple RCVS Knowledge channels including a press release, newsletters, and social media.

The target sample size of 381 was calculated based on an estimated population of 35,000 to 37,000 veterinary surgeons and RVNs working in small animal or mixed practices in the UK, in order to achieve a 95% confidence level and a 5% margin of error. Sample size and composition was monitored against a sampling framework based on RCVS data to ensure that a range of practice types, job roles, and experience were represented (RCVS, 2024; Rosolin et al., 2024). A heavier weighting was given to veterinary surgeons due to their central role in clinical decision-making. Targeted recruitment helped address the initial underrepresentation of RVNs, professionals from charity settings, and recent graduates, though the final sample included a slight overrepresentation (3%) of early-career veterinary surgeons.

The inclusion criteria for the pet owner survey were current ownership of at least one dog and/or cat and having visited a veterinary practice in the UK within the past three years. Exclusion criteria were being under 18 years of age and residing outside the UK. The survey used convenience sampling, recruiting participants through Kaleidoscope’s professional networks and organic social media posts on Facebook. After two weeks of data collection, response rates were below the target of 381. Recruitment was enhanced through paid Facebook advertising targeting UK pet owners, supplemented by addition of an incentive prize draw (£100 voucher randomly awarded to one participant who provided contact information).

Fifteen semi-structured 30-minute online interviews were conducted in April and May 2025. Participants were recruited through purposive sampling to ensure diversity in role, experience, and practice type. Participants included veterinary surgeons, RVNs, practice managers and receptionists from various practice settings (corporate, independent, charity), and veterinary educators.

Two 1-hour focus groups were conducted online via Microsoft Teams in May 2025. Ten dog and cat owners were recruited from survey participants who expressed interest in further involvement. Participants received £25 gift vouchers as compensation for their time. Interviews and focus groups were audio-recorded and transcribed with participant consent.

#### Data analysis

The quantitative survey data were cleaned and analysed using PSPP version 2.0. Frequencies, percentages, means, and standard deviations were calculated as appropriate. Chi-square tests were used to examine differences in nominal variables (e.g. barrier selection, professional role, practice ownership type). Mann–Whitney U and Kruskal–Wallis tests were used to examine ordinal variables in Likert-scale responses, as appropriate to the number of comparison groups. This non-parametric approach was used given the discrete, ordered response categories and distribution characteristics typical of attitudinal data.

Statistical significance was set at α = 0.05. Bonferroni correction was applied for multiple comparisons within each analysis family to control family-wise error rate. Where expected cell counts were insufficient for robust chi-square analysis (n < 5), subgroups were excluded from comparative testing to avoid violations of test assumptions.

Where statistically significant differences were detected using chi-square or Kruskal–Wallis tests, post-hoc pairwise comparisons were not conducted. Therefore, while we report significant variation among subgroups, we describe patterns in the data rather than identifying specific pairwise differences.

The qualitative data from interview and focus groups transcripts were coded by a team of three researchers using an initial coding framework developed deductively from the research framework and research questions. Additional inductive codes were developed during analysis as new themes emerged from the data. Discrepancies were discussed and resolved through consensus.

Open-text responses from both surveys were initially coded using artificial intelligence platforms (Claude Sonnet 4 and Gemini 2.5 ProPreview). The results were cross-checked and all survey responses reviewed in full twice by researchers, who revised the AI-suggested themes as needed.

For analysis of open-text responses of the pet owner data, the research team employed quota sampling to select a qualitative analysis sample of 385 from the full quantitative sample of 718. The quota sampling strategy prioritised inclusion of underrepresented groups (men, non-binary respondents, ethnic minorities, and participants under 55 years) to offset the overall sample skew toward women, white ethnicity, and older age groups. All open-text responses from the 417 veterinary professional survey participants were included in qualitative analysis.

Results were compared across data sources and participants to identify areas of convergence, complementarity, and discrepancy.

## Results

### Demographics

The survey of veterinary professionals achieved an initial sample of 418 participants. During data cleaning, one participant was excluded due to straight-lining responses, resulting in a final sample of 417 participants, made up of 313 veterinary surgeons (75%) and 104 RVNs (25%). This sample was predominantly female (83%), with 15% male and 2% selecting other options. Experience levels among veterinary surgeons were distributed across graduation cohorts: 28% qualified since 2020, 45% qualified between 2000–2019, and 27% qualified before 2000. Among RVNs, 67% had 10 or more years of experience. The sample consisted of 52% working in corporate practices, 33% in independent practices, 13% in charity practices, and 8% in other practice types (percentages exceed 100% as respondents could select multiple categories). The final sample achieved a 95% confidence level with a 5% margin of error when compared to RCVS workforce data, with a 3% overrepresentation of early career veterinary surgeons. The majority (65%) described themselves as working in first opinion practice, 10% in Emergency/Out of Hours provision, 14% in Veterinary Hospitals, 3% in university practice, 8% in referral practice, and 2% in charity practice (percentages exceed 100% as respondents could select multiple categories).

The survey of pet owners resulted in 763 completed responses. Application of inclusion and exclusion criteria led to exclusion of 45 participants, resulting in a final sample of 718. The pet owner sample was majority female (71%), white ethnicity (76%), and older age groups (58% aged 55+). The majority of the sample (87%) had owned a dog and/or cat for six years or more, while 5% had between three and six years of ownership experience, 3% had less than 3 years’ experience and 5% were breeders. Pet insurance coverage among the sample was split, with 44% having no insurance, 44% having insurance for all their cats and/or dogs and 12% having insurance for some, but not all, of their cats and/or dogs.

### Perceived barriers to contextualised care – veterinary professionals

**Figure 1:**
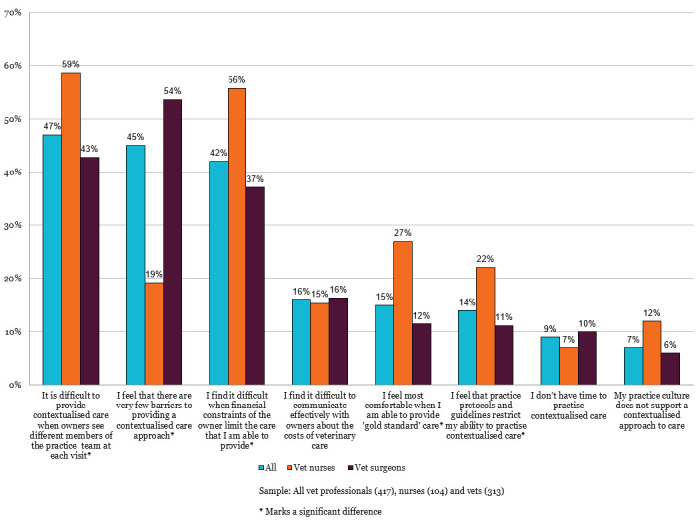
The biggest barriers to providing contextualised care (up to three selected), shown by role (RCVS Knowledge 2025b).

In response to the question in which veterinary professionals were asked to select the three biggest barriers to providing contextualised care, the most frequently selected barriers were lack of continuity of care (47%) and owner financial constraints limiting the care they could provide (42%), while 45% of respondents selected that they experience very few barriers to providing contextualised care. However, a significantly higher proportion of veterinary surgeons than RVNs reported experiencing very few barriers. RVNs consistently reported higher rates of barriers across most categories, with statistically significant differences for continuity of care challenges, financial constraints, feeling most comfortable when providing 'gold standard' care, and feeling restricted by practice protocols and guidelines (Figure 1).

**Figure 2:**
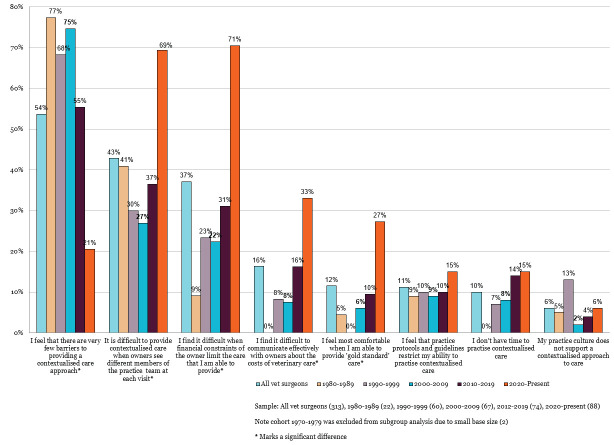
The biggest barriers to providing contextualised care (up to three selected), shown for veterinary surgeons only, by year of qualification (RCVS Knowledge 2025b).

Differences also emerged among veterinary surgeons when analysing perceived barriers by years since qualification. Those graduating since 2020 reported substantially higher rates across most barriers and lower rates of experiencing very few barriers than all other cohorts. These differences were most marked for continuity of care challenges, financial constraints, communicating about costs, and feeling most comfortable providing 'gold standard' care (Figure 2).

Differences by time since graduation were also found in responses to the question ‘I tend to rely on tests and diagnostics to get a clear answer because I am worried about client complaints or disciplinary action if I make the “wrong” decision’, with just 9% among those qualified in the 1980s agreeing or strongly agreeing with the statement compared to 50% among those qualified since 2020.

Similarly, feeling relieved when pets are insured increased from 36% to 83% across these same cohorts. Conversely, confidence in providing appropriate care without a definitive diagnosis decreased from 92% among veterinary surgeons who qualified before 2010 to 66% among those who qualified since 2020.

The qualitative data helps to illuminate the reasons for this.

‘I think contextualised care is more difficult for younger vets because they are frightened they are going to miss things. A younger vet will do a lot more blood testing than I would because they haven't got that experience yet’ (Veterinary surgeon).

‘New grads feel so overwhelmed, they fear the RCVS is going to look at them, or owners are, they are going to get complaints’ (RVN).

‘I feel that they [new graduates] are really good at knowing what the options are, and they're really good for treatment, knowing what the best one is for that condition in isolation but I think they lack confidence in being able to make a call to say ‘I think this is the best for you’. Because I think that's a clinical confidence as well as a communication confidence’ (Veterinary educator).

**Figure 3:**
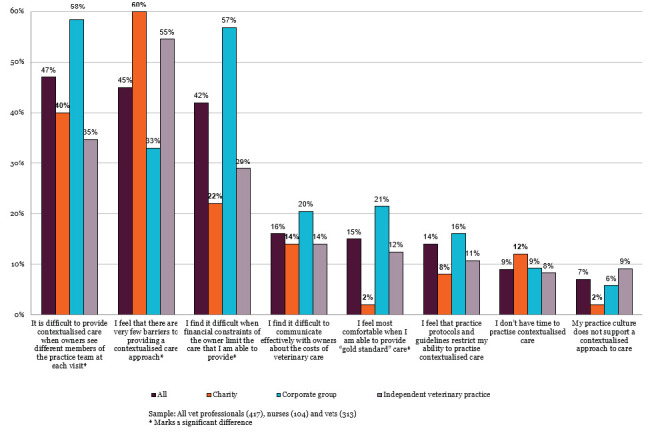
The biggest barriers to providing contextualised care (up to three selected), shown by practice type (RCVS Knowledge 2025b).

Differences in perceived barriers were also identified by practice type, with continuity of care, owner financial constraints, and feeling most comfortable when able to provide 'gold standard' care selected as barriers to the delivery of contextualised care at significantly higher rates by veterinary professionals working in corporate practice than those working in independent practice or charity settings (Figure 3).

Higher percentages of veterinary surgeons in corporate practice also reported relief when pets are insured (77% vs 55% independent, 27% charity), and fewer reported confidence in providing care without definitive diagnosis (72% vs 89% independent, 95% charity).

Pet owners expressed concerns that vets may be under pressure to follow standardised protocols which they perceive as a barrier to providing contextualised care.

‘Newly trained vets seem like they're going through a set protocol which will direct you into certain treatments. This worries me. It's difficult finding a vet with all the experience who can look at an animal and think yes it doesn't need surgery’ (Pet owner).

‘When it comes to personalised care, it has a lot to do with the vet… the amount of pressure, targets, facilities, if they are focusing on one species or multiple’ (Pet owner).

### Lack of continuity of care

Lack of continuity of care was perceived as a major barrier to the delivery of contextualised care, with 47% of veterinary professionals selecting ‘It is difficult to provide contextualised care when owners see different members of the practice team at each visit.’

Pet owners also strongly valued continuity of care, with 78% agreeing or strongly agreeing that it is important to see the same veterinary team members when visiting the practice.

The qualitative data provides a deeper understanding of why lack of continuity of care creates a barrier to the provision of contextualised care, highlighting concerns about capturing and sharing information in the practice:

‘The common complaint is “I always see a different person every time I go in.” They're having to start the story from scratch, and sometimes you get duplication of testing because the next vet isn't fully aware of what's gone on before or even the thinking of the previous day’ (Veterinary surgeon).

‘Lack of continuity of care [is a barrier]. We still don't have a good culture of the same vet seeing the same owner and we aren't always good about recording details about the client because we are nervous about including personal details in the notes’ (RVN).

The challenge extended beyond simple information transfer to encompass the importance of building relationships in order to provide continuity of care:

‘I think it's easiest to deliver [contextualised care] if you have a good relationship with the client but unfortunately [clients] rarely see the same doctor because they are working part time and it's hard to get an appointment with them. It's so much easier [when] you get to know the client's approach. You get to know the client's willingness to do things... if you have that relationship it's much easier to discuss the case in depth then to find the appropriate route’ (Veterinary surgeon).

‘Seeing a different vet every time you visit [means the] vet has no personal relationship with me or with the dog, therefore carries no direct knowledge of me or of the dog or the dog's history’ (Pet owner).

The consequences of lack of continuity of care can lead to pet owners losing confidence in the advice provided and negatively impacting the relationship and trust between the pet owner and the veterinary team:

‘Different vets at the clinic might give different advice or options for treating my dog which can be confusing and make it harder to choose the right option for my dog’ (Pet owner).

‘At my current vet practice, there's been a lot of changeover of vets. This means my pet is seen by a different vet each time, and that makes it challenging for anyone to develop a relationship or trust. Often, different vets give different advice as well, which means we're getting conflicting information’ (Pet owner).

‘I feel that the vet team often don't agree with and therefore ignore/dismiss information I've given them’ (Pet owner).

‘Doing my own research to understand conditions and medications along with side effects and bringing up this in consultations [is] met with hostility when I seem to know more or ask deeper questions about things pertaining to my dogs' illnesses’ (Pet owner).

### Financial constraints

Financial constraints were perceived as a major barrier to the delivery of contextualised care, with 42% of veterinary professionals selecting ‘I find it difficult when financial constraints of the owner limit the care that I am able to provide.’ The percentage was higher for RVNs than veterinary surgeons (56% v 37%, Figure 1), veterinary surgeons that qualified since 2020 (71%, Figure 2), and veterinary professionals working in corporately owned practices (57%, Figure 3).

When veterinary professionals were asked how often owner financial constraints influenced their care of individual patients 47% responded always, 43% often, 9% sometimes, and only 1% rarely.

The qualitative data indicates that veterinary professionals experience tensions between providing optimal care and managing the financial concerns and limitations of pet owners:

‘It is sometimes difficult for owners to understand/accept that financial constraints may limit the care that is available to their pet’ (Veterinary surgeon).

‘Owners not accepting their own financial limitations, i.e. offering cheaper options, but owners becoming aggressive and blaming staff for the fact that it's not possible for the patient to receive “gold standard” treatment’ (Veterinary surgeon).

‘Due to the current economic climate, we've seen more patients be euthanised or [owners] wait to bring their pet in for care due to financial concerns, so by the time we see someone they may be much worse off than they could've been’ (RVN & practice manager).

There are also indications that some veterinary professionals feel commercial pressure to generate income for the practice.

‘I am in the position of practising [contextualised care] in my full-time job and am very comfy with it but find my locum work really hard as I work for corporates that want to push everything at a client even when I don't feel it is needed’ (Veterinary surgeon).

‘I feel some clinics may be incentivised to increase client spend in order to repay large equipment purchases such as MRI/CT’ (RVN).

‘In the corporates, it is about bringing in the money. I know that there is pressure on my manager to bring in money, what more could we be doing. We have a team meeting this afternoon before the shift starts and that will be an uncomfortable conversation for me because I will still not be recommending things that are unnecessary. So probably one barrier is organisational financial goals and priorities. And they are businesses. I get that, they have to run, they have to pay their staff, I understand that’ (Veterinary surgeon).

**Figure 4:**
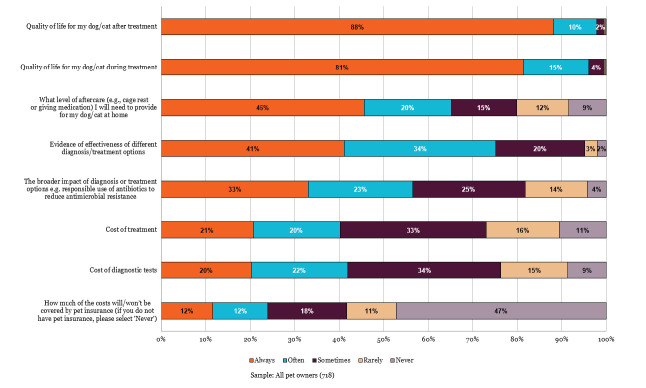
Factors considered by dog and cat owners when making choices about diagnostic and treatment options (RCVS Knowledge 2025b).

Owners report that they consider the costs of veterinary care less often than concerns around quality of life and evidence of effectiveness (Figure 4). However, when asked about barriers to contextualised care, 31% of pet owners selected feeling guilty when they cannot afford all the options on offer (Figure 6).

When asked about satisfaction with aspects of care received over the past three years, only 42% of pet owners felt satisfied that veterinary teams considered their financial situation when making recommendations, with 24% expressing dissatisfaction.

The qualitative data also indicates that the costs of veterinary care can be a barrier.

‘Treatments are often tailored to the dog but these treatments can be extremely expensive and not covered through insurance e.g. canine behaviourist’ (Pet owner).

‘Just costs [are the barrier], especially since vets are now not allowed to advise human medicines for pets, meaning the licenced alternative is hugely expensive’ (Pet owner).

‘[The] biggest problem for me is the ridiculous cost of out of hours veterinary care. My own vets are great but not open at weekends. The out of hours service is run by a different company and the cost is extortionate’ (Pet owner).

Communication about costs emerged as a specific challenge for both veterinary professionals and pet owners. Whilst only 16% of veterinary professional respondents overall selected difficulty communicating effectively with owners about costs as one of their top three barriers, this figure varied substantially by experience, with 33% of recent graduates (2020–present) identifying this as a barrier compared to 0% of those qualified in the 1980s (Figure 2).

The qualitative data revealed the complexity of discussing costs sensitively.

‘The vets that do [cost communication] well literally provide estimates for the client and it's all done in the appointment. One of the vets is the best at it. And you do hear her saying “gold standard would be this, but we can also try this and there is no harm in trying this first” so the client doesn't feel bad if they can't afford gold standard. So, she's brilliant but it's about getting that level taught out to all other vets’ (Practice manager).

‘Sometimes owners also feel upset when you offer gold star care simply because it would feel dishonest not to offer even if you are happy to work with them. I've been accused of “shaming” owners for not being able to afford the gold star option even when I'm very happy to adjust’ (RVN).

From the pet owner perspective, lack of transparent cost communication from veterinary practices emerged as a significant concern.

‘The vets always assume money is no object and I am not usually told the costs (e.g. for blood tests) in advance’ (Pet owner).

‘The offers we received were mostly best for the practice, not for the pet. I strongly believe that tailoring the best possible options for a certain pet is a very good approach. However, owners who cannot fully afford more expensive treatment should be informed of the [other options]’ (Pet owner).

‘When my vet went from being independent and sold out to a big company, suddenly they didn't want to talk money, rather it's we need to do this test, or this test ... I'm currently with an independent vet who is open and happy to talk about money ... It needs to be not a taboo topic, honest and open from the beginning so it's not a shock at the end’ (Pet owner).

**Figure 5:**
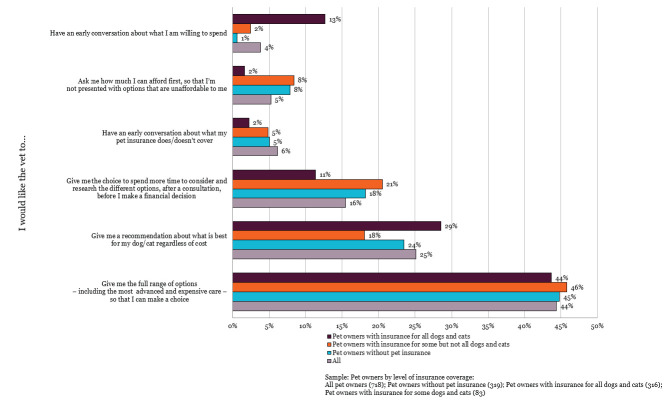
Dog and cat owners' preferences for discussion of costs (RCVS Knowledge 2025b).

However, when asked about how they would like their vet to inform them of the costs of treatment the results show that very few pet owners want to be asked directly about how much they are willing or able to spend (Figure 5). Instead, the top option (selected by 44% of respondents) was to be given the full range of options to enable them to make a choice. The next most popular option, selected by 25% of the sample, was to be given a recommendation about what is best for the pet regardless of cost. These results did not differ significantly with the level of insurance cover.

### Defensive medicine

Survey respondents articulated how fear may prevent veterinary professionals from using clinical judgement to tailor care appropriately. Veterinary surgeons responding to open-text survey questions said:

‘I find that vets are often so scared of stepping out of a box that they don't even consider an alternative approach’ (Veterinary surgeon).

‘So many vets are terrified of disciplinary action that this informs how they practice’ (Veterinary surgeon).

‘I think you would find there is a lot of anxiety out there. I try to be pragmatic but 100% there are nights when I come home obsessing over whether I should have done this or that, did I write that properly, should I have offered this? That is all emotional mental load’ (Veterinary surgeon).

‘It isn't just a mental health approach but it's building resilience within our veterinary population, to help them understand that change is okay and not having gold standard is okay. Also helping them to deal with unhappy clients and emotional clients. Because I think there is a lot of defensiveness over that’ (Veterinary surgeon).

**Figure 6:**
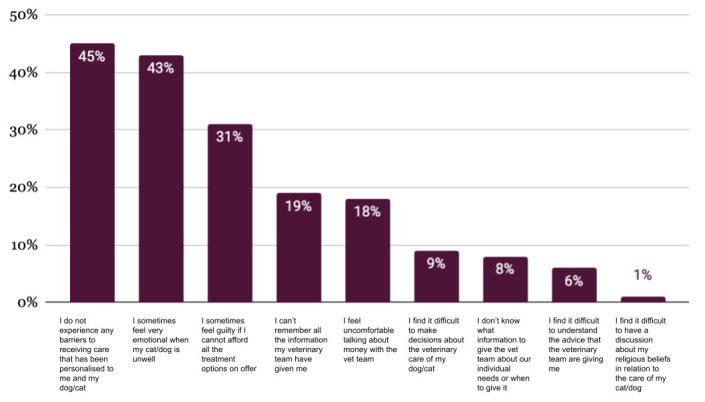
The biggest barriers to receiving contextualised care, identified by pet owners (up to three selected) (RCVS Knowledge 2025b).

Pet owners also acknowledge the impact emotions can have. When asked to choose the three statements that best reflect the barriers they experienced in receiving personalised care, 43% selected feeling very emotional when pets are unwell and 31% sometimes feeling guilty about not being able to afford all the treatment options on offer (Figure 6).

## Discussion

A positive finding of this study is that 45% of veterinary professionals and pet owners report that they encounter very few barriers in providing or receiving contextualised care, confirming that this is something that is achievable in practice. However, this research has identified a number of perceived barriers to contextualised care.

Lack of continuity of care was perceived as a significant barrier to the delivery of contextualised care by both veterinary professionals and pet owners. In human medicine continuity of care has been shown to be associated with both improved clinical outcomes and patient satisfaction, particularly for those with chronic conditions (Cabana & Jee, 2004; Van Walraven et al*.*, 2010).

In this study, pet owners associated continuity of care with building trusting relationships and ensuring consistent care. Inconsistency of care and lack of trust have been reported to be associated with reduced perception of value and increased likelihood of switching practice (Brown, 2018; Hickey et al*.*, 2024). Veterinary professionals also acknowledge these concerns. In addition, they identified the ability to capture and share information within the practice team as a barrier to the delivery of contextualised care, highlighting concerns about recording contextual information about the owner in the patient notes.

There are a number of reasons why continuity of care may be more difficult to deliver now than it was in the past. Changes in the delivery of veterinary services over the last twenty years have seen significant increases in the use of out of hours service providers and referral services, meaning that pet owners may visit several different practices with their animal, even during the course of a single condition.  There has also been a significant increase in flexible and part-time working, meaning that it may be more difficult for pet owners to see the same members of the veterinary team, even at the same practice (Kinnison et al., 2014; Rosolin et al., 2024).

It may therefore be necessary for veterinary practices to consider whether continuity of care can be delivered at a team level. This will require contextual information to be captured and shared between members of the practice team. Recording any information about the client will need to be done with client consent and in a way that is compliant with data protection regulations, which may require guidance from professional bodies. However, it has been noted that the delivery of care by multidisciplinary teams and the expansion of digital and remote technologies have created both opportunities and challenges for continuity of care (Freeman & Hughes, 2010; Pyatt et al*.*, 2020).

The costs of veterinary care have risen significantly in recent years with the costs of veterinary and other pet services in the UK reported to have increased by over 75% since 2015 (Office for National Statistics, 2025), with significant rises also being reported in other countries (Egenvall et al*.*, 2024).

The reasons for these increases are complex, but include advances in diagnostic and treatment options, changes in owner expectations, increased popularity of pets with exaggerated conformation, as well as changes in the delivery of veterinary services (O’Neill et al*.*, 2023; CMA, 2024; Corr et al*.*, 2024). However, whatever the reasons, the increase in the costs of veterinary care has led to issues of affordability for pet owners and potential adverse welfare outcomes for pets (Croney et al., 2025; O’Connor et al*.*, 2025) .

The affordability of veterinary services also impacts the wellbeing of veterinary professionals (Kipperman et al., 2017; Williams, 2025), with financial constraints influencing the care of individual patients and a recent survey of UK veterinary professionals showing that the affordability of veterinary services was rated as the third greatest challenge facing the veterinary profession (46%) after client expectations/demands (54%) and stress levels (49%) (Rosolin et al., 2024).

Although contextualised care, in terms of adapting care to the individual circumstances of the owner, may be seen as a potential way to mitigate financial constraints, this research shows that there are still barriers relating to having open conversations about the costs of veterinary care.

The survey data showed that when owners are asked how they would like their vet to let them know about the potential costs of treatment, the most frequently selected options were to be given the full range of options to enable them to make a choice or to be given a recommendation about what is best for the pet regardless of cost. However, less than 10% wanted to be asked directly about how much they can afford and even fewer wanted to have an early conversation about what their insurance covers. Despite veterinary surgeons reporting relief when animals are insured, owner preferences regarding the discussion of costs do not appear to vary in line with level of insurance, suggesting that other contextual factors are important.

These findings provide some evidence as to why discussions of the cost in veterinary consultations may perceived as challenging and the need for these discussions to move from discussion of tangibles, such as time and services, to outcomes related to the pet's health and well-being (Coe et al., 2007, 2009; Groves et al., 2022).

The impact of owners not being able to afford veterinary care has an impact on the welfare of animals and the wellbeing of pet owners and veterinary professionals. Many pet owners in this study report sometimes feeling very emotional when their animal is unwell and feeling guilty if they cannot afford all the treatment options on offer. These findings are supported by other research that suggests that decisions regarding an animal’s care are impacted by the owner’s emotional attachment to the animal rather purely financial concerns (Brown, 2018; Corr et al., 2024; Strand et al*.*, 2024) which may lead to conflict between the veterinary care that they can afford and the care they would like their pet to receive.

There is also evidence that the high costs of veterinary care can impact the wellbeing of veterinary professionals, and that recently qualified vets in particular may feel unprepared to deal with financial constraints that impact their ability to treat animals to their preferred standard or necessitate having to perform euthanasia when treatment is unaffordable (Kipperman et al., 2017; Williams, 2025).

Alongside age and experience, there is evidence that professional identity can impact the response to contextual challenges. Two distinct variants of veterinary identity have been proposed: ‘diagnosis-focused’ identity, which prioritises definitive diagnosis and best-evidence treatment; and ‘challenge-focused’ identity, where priorities were also reported to include engaging with the client, and contextual challenges including limited finances (Armitage‐Chan & May, 2018). It may be that those with a challenge-focused identity find it easier to practise contextualised care.

The anxiety around disciplinary action documented in responses suggests that there is a perception that current professional standards discourage the clinical flexibility required for contextualised care, even though the Royal College of Veterinary Surgeons (RCVS) has recently updated their guidance to explicitly make reference to providing contextualised care (RCVS, 2025).

The fear of regulatory scrutiny, missed or incorrect diagnoses, and client complaints may be contributing to a defensive approach to practice in which additional tests or treatments that are of little or no benefit to the patient are carried out, raising the costs of veterinary care and acting as an additional barrier to the delivery of contextualised care (Summerton, 2000; Assing Hvidt et al., 2017; Gibson et al., 2022),

This study detected several differences in the perceived importance of barriers to contextualised care by experience, role, and type of practice. It is not surprising that those that have graduated more recently generally perceive greater barriers to the delivery of contextualised care as they have less experience to draw on. However, to ensure that they have the knowledge and skills to deliver contextualised care it is important that they are not only exposed to a range of evidence-based treatment options but have the people and communication skills to work with pet owners in potentially emotionally charged situations, to find the most appropriate option.

While changes have already been made in the UK to ensure that the majority of clinical education must focus upon casework in the ‘general practice’ context (RCVS, 2023), it is important to ensure that ideas relating to professional identity, or a single most appropriate approach, are not instilled through the hidden curriculum (Roder & May, 2017; Kunze & Seals, 2020).

The American Association of Veterinary Medical Colleges have recently published an implementation strategies guide to prepare graduates to deliver contextually appropriate, evidence-based care across a range of clinical settings, indicating that these concerns are not limited to the UK (Fedesco et al*.*, 2025).

This research also found that RVNs consistently perceived greater barriers to the delivery of contextualised care across most categories. Since our sample of RVN respondents was skewed towards those with greater experience (> 10 years), it is more likely that this finding relates to differences in role rather than experience. Going forward it will be essential to ensure that veterinary nurses also have the knowledge and skills to support and deliver contextualised care.

While the quantitative data indicates that those working in corporate practices were more likely to report a range of barriers to contextualised care, interpretation of these findings is complicated by the fact that nearly half (48%) of corporate practice respondents in the sample qualified in the last five years, compared with 15% of respondents working in independent practice, suggesting years of experience may be a confounding factor.

However, it is also important to recognise that the qualitative data reveals a clear perception among both veterinary professionals and pet owners that corporate ownership is associated with a range of barriers to contextualised care, and there is evidence to support this from other areas where private equity has become involved in the delivery of healthcare (Singh et al., 2022; Bruch et al., 2023; Zhu et al., 2024).

This study has both strengths and weaknesses. This is the first study of contextualised care to collect and analyse data from veterinary professionals and dog and cat owners. The involvement of stakeholders at two meetings, before and after data collection, enabled us to incorporate a wide range of perspectives both into the framing of the questions and providing feedback on the findings to develop actionable recommendations (RCVS Knowledge, 2025b). One disadvantage of this approach is that we asked a wide range of questions, and with hindsight focusing on a narrower range of topics in more depth might have given more robust results. However, this research now provides a broad foundation for future work. The questionnaires were completed by a self-selecting sample, and therefore the responses were subject to selection bias. While it was possible to compare the representativeness of the veterinary professional data, at least in terms of certain criteria, against RCVS workforce data, any assessment of the representativeness of the pet owner data is limited by the absence of published demographic data for the UK veterinary-visiting pet owner population, although there is evidence from other veterinary questionnaires that respondents to veterinary questionnaires are primarily female (Belshaw et al., 2025; Glasspool et al., 2025).

The data for this study were collected over a concentrated period (April–July 2025), capturing a snapshot of contextualised care during this timeframe. As this coincided with the CMA investigation in veterinary services in domestic pets, the discourse around and media coverage of this investigation may have influenced the responses of both veterinary professionals and pet owners.

While this paper reports on the most frequently reported barriers to the delivery of contextualised care it should be remembered that for certain individuals, other factors such as access to transportation, language, and cultural differences may be of great significance.

The questionnaires were limited to responses from veterinary surgeons and RVNs, and dog and cat owners respectively. However, it is reasonable to assume that the principles of contextualised care, and adapting the care provided to the individual circumstances and context in which care is delivered, will be relevant to all members of the veterinary team including those working in other contexts including farm, equine, and exotic practice, as well as with wildlife.

### Conclusion

This paper presents the first empirical evidence on the barriers to contextualised care as perceived by both veterinary professionals and dog and cat owners.

The main barriers to contextualised care identified were lack of continuity of care and financial constraints. Continuity of care is important to the delivery of contextualised care as it enables a trusting relationship to develop and for contextual information, including the owner’s personal values and circumstances, to be shared alongside clinical information. While financial constraints were identified as a barrier to contextualised care, they are more accurately characterised as an important contextual factor. However, the problems identified around discussing the costs of care do act as a barrier to the delivery of contextualised care.

A strong theme that emerged in this study was the impact of emotions on the veterinary consultation, with veterinary professionals identifying fear of regulatory scrutiny, complaints, and clinical failure as barriers to implementing contextualised care, and with pet owners reporting that they feel emotional when pets are unwell and feel guilty about not being able to afford treatment.

This research identified a number of differences in the barriers veterinary professionals perceived to contextualised care based on their level of experience, role in practice, and type of practice in which they work. It is therefore important to recognise that the barriers to contextualised care may be felt differently by individuals for a range of reasons, and to ensure that all members of the practice team are supported to understand the context in which they are working to deliver contextualised care.

This research was limited to responses from veterinary professionals working in small animal practice, and dog and cat owners respectively. However, the principles of contextualised care will be relevant to those working in other contexts including farm, equine, and exotic practice, as well as with wildlife.

To overcome the barriers to contextualised care it will be important to ensure that those working in veterinary practice receive support to develop the knowledge and skills to deliver contextualised care. This will require support at all levels, including from professional bodies, educators and those with leadership positions in veterinary practice (RCVS Knowledge, 2025a).

## Ethical approval

RCVS Ethics review panel 2025-115.

## Informed consent

Informed consent for use of anonymised data was obtained from all participants as part of the recruitment process.

## Author contributions

**Sally Everitt:** Conceptualisation, Visualization, Writing – Original draft. **Andréa Taylor:** Methodology (lead), Formal analysis (lead), investigation, Writing – Review & Editing. **Ashley Doorly:** Project Administration, Visualization, Writing – Review & Editing. **Katherine Cropley:** Methodology (supporting), Formal Analysis (Supporting,) Investigation. **Allison Osterberg:** Methodology (Supporting), Formal Analysis (Supporting,) Investigation. **Katie Mantell:** Funding Acquisition, Writing – Review & Editing.

## Acknowledgements

Thanks to Naheem Bashir (Kaleidoscope Health and Care) for his expert advice and review in the data set up and analysis phases.

## Funding

Battersea supported this research through their grants programme but they were not involved in the research and did have any input into the findings or the recommendations.

## ORCiD

Sally Everitt: https://orcid.org/0009-0009-7739-7134

Andréa Taylor: https://orcid.org/0000-0002-3684-3312

Ashley Doorly: https://orcid.org/0000-0003-3336-1665

Katherine Cropley: https://orcid.org/0009-0008-3168-8457

Allison Osterberg: https://orcid.org/0009-0009-7945-8869

Katie Mantell: https://orcid.org/0000-0002-1292-5014

## Conflict of Interest

Sally Everitt, Ashley Doorly, and Katie Mantell are employees of RCVS Knowledge. RCVS Knowledge received financial support for this study from Battersea but they were not involved in the research and did have any input into the findings or the recommendations. Andréa Taylor, Katherine Cropley, and Allison Osterberg are employees of Kaleidoscope Health and Care. Kaleidoscope was contracted and paid by RCVS Knowledge to conduct the research presented in this article.

## Disclaimer

Any opinions expressed in articles and other publication types published in Veterinary Evidence are the author's own and do not necessarily reflect the view of the RCVS Knowledge. Veterinary Evidence is a resource to help inform, and the content herein should not override the responsibility of the practitioner. Practitioners should also consider factors such as individual clinical expertise and judgement along with patient’s circumstances and owners’ values. Authors are responsible for the accuracy of the content. While the Editor and Publisher believe that all content herein are in accord with current recommendations and practice at the time of publication, they accept no legal responsibility for any errors or omissions, and make no warranty, express or implied, with respect to material contained within. For further information please refer to our Terms of Use.
